# Induction with Lopinavir-Based Treatment Followed by Switch to Nevirapine-Based Regimen versus Non-Nucleoside Reverse Transcriptase Inhibitors-Based Treatment for First Line Antiretroviral Therapy in HIV Infected Children Three Years and Older

**DOI:** 10.1371/journal.pone.0108063

**Published:** 2014-09-18

**Authors:** Gerardo Alvarez-Uria, Raghavakalyan Pakam, Praveen Kumar Naik, Manoranjan Midde

**Affiliations:** Department of Infectious Diseases, Rural Development Trust Hospital, Bathalapalli, AP, India; University of Pittsburgh, United States of America

## Abstract

**Background:**

The World Health Organization recommends non-nucleoside reverse transcriptase inhibitors (NNRTIs)-based antiretroviral therapy (ART) for children three years and older. In younger children, starting ART with lopinavir boosted with ritonavir (LPVr) results in lower risk of virological failure, but data in children three years and older are scarce, and long-term ART with LPVr is problematic in resource-poor settings.

**Methodology:**

Retrospective cohort of children three years and older who started triple ART including LPVr or a NNRTI between 2007 and 2013 in a rural setting in India. Children who started LPVr were switched to nevirapine-based ART after virological suppression. We analysed two outcomes, virological suppression (HIV-RNA <400 copies/ml) within one year of ART using logistic regression, and time to virological failure (HIV-RNA >1000 copies/ml) after virological suppression using Cox proportional hazard regression. A sensitivity analysis was performed using inverse probability of treatment weighting (IPTW) based of propensity score methods.

**Findings:**

Of 325 children having a viral load during the first year of ART, 74/83 (89.2%) in the LPVr group achieved virological suppression *versus* 185/242 (76.5%) in the NNRTI group. In a multivariable analysis, the use of LPVr-based ART was associated with higher probability of virological suppression (adjusted odds ratio 3.19, 95% confidence interval [CI] 1.11–9.13). After IPTW, the estimated risk difference was 12.2% (95% CI, 2.9–21.5). In a multivariable analysis including 292 children who had virological suppression and available viral loads after one year of ART, children switched from LPVr to nevirapine did not have significant higher risk of virological failure (adjusted hazard ratio 1.18, 95% CI 0.36–3.81).

**Conclusions:**

In a cohort of HIV infected children three years and older in a resource-limited setting, an LPVr induction- nevirapine maintenance strategy resulted in more initial virological suppression and similar incidence of virological failure after initial virological suppression than NNRTI-based regimens.

## Introduction

Due to higher viral loads, pharmacokinetic variability and suboptimal adherence because of complex regimens and frequent dosing adjustments, suppression of viral replication after initiation of antiretroviral therapy (ART) is more difficult to achieve in children than in adults [1]. Compared with non-nucleoside reverse transcriptase inhibitors (NNRTIs), protease inhibitors (PIs) have a higher genetic barrier [2]. Studies performed in adults in resource-limited settings show that boosted PI-based regimens result in less NRTI resistances [3], [4]. In children <3 years, randomized control trials have demonstrated that the use of lopinavir boosted with ritonavir (LPVr) based regimens has lower risk of virological failure than ART containing nevirapine (NVP) [5], [6]. In children >3 years, observational studies in resource-limited setting suggest that the use of PI-based ART is also associated with lower risk of virological failure, but data are scarce [7], [8].

Once virological suppression is achieved, the risk of virological failure of ART with NNRTIs is considerably reduced [5], [9]. In children taking PI-based regimens, switching to a NNRTI-based regimen after virological suppression can result in multiple benefits, such as alignment with adult ART, lower cost, better metabolic profile, simplification of ART with fixed-dose combinations, and preserving PIs for second line treatment [10]. In this study, we compared the virological response of children who received NNRTI-based ART with those treated with LPVr-based ART followed by switch to NVP-based treatment in an HIV cohort study in India.

## Methods

This study was approved by the Ethical Committee of the Rural Development Trust (RDT) Hospital. Written informed consent was given by caretakers for their information to be stored in the study database and used for research.

### Setting

The study was performed in Anantapur, a district situated in the South border of Andhra Pradesh, India. Anantapur has a population of approximately four million people, and 72% of them live in rural areas [11]. RDT is a non-governmental organization that provides medical care to HIV infected people free of charge, including medicines, consultations, and hospital admission charges. In our setting, the HIV epidemic is largely driven by heterosexual transmission and it is characterized by poor socio-economic conditions and high levels of illiteracy [12]. Although the vast majority of children acquire HIV perinatally, 8% of female children acquire HIV through sexual contacts and 90% of them are diagnosed after aged 18 months. Near half of children have lost one or both of their parents [12].

### Study design and definitions of variables of interest

The Vicente Ferrer HIV Cohort Study (VFHCS) is an open cohort study of all HIV infected patients who have attended Bathalapalli RDT Hospital. The baseline characteristics of the cohort have been described in detail elsewhere [12], [13]. The cohort is fairly representative of the HIV population in the district, as it covers approximately 70% of all HIV infected people registered in the district [14]. For this study, we selected HIV infected children aged 3 to 16 years who started first-line ART from January 1^st^ 2007 to December 31^st^ 2013 from the VFHCS database. The selection of patients from the database was executed on June 14^th^ 2014 (end of the follow-up period).

ART was started by clinical criteria (clinical stage 3 or 4 of the World Health Organization [WHO]) or by immunological criteria (CD4 count <750 cells/µl or <20% in children aged 36–59 months, and CD4 count <350 cells/µl in children aged >59 months) according to the Indian National Guidelines [15]. Children started ART with two NRTIs and a NNRTI (NVP or efavirenz) or LPVr. Children who started LPVr were switched to a NVP-based regimen after achieving virological suppression. To facilitate dose calculations, we used weight band-based dosing of paediatric LPVr tablets (100 mg/25 mg) to achieve an approximate dose of 300 mg/m^2^/dose (10–20 kg, 2-0-2; 20–30 kg, 3-0-3; >30 kg, 4-0-4) [16], [17]. Paediatric LPVr tablets were substituted by larger adult tablets (200 mg/50 mg) if the child was able to swallow them (in these cases, the dosing in the 20–30 kg band was 2-0-1 adult tablets). Caretakers and children were instructed not to break LPVr tablets. We did not use liquid formulation of LPVr due to its poorer palatability, and cold-chain requirements. Other anti-retroviral drugs were given as per WHO and National guidelines [18].

Viral load was performed every six months after ART initiation. High viral load at baseline was defined as having >100,000 copies/ml of HIV-RNA [19]. Initial virological suppression was defined as having <400 copies/ml of HIV-RNA during the first year of ART. Following the WHO definition, virological failure was defined as having >1,000 copies/ml of HIV-RNA after six months of ART in two consecutive viral load determinations [20]. However, children having a single viral load >1,000 copies/ml only in the last viral load determination were considered to have virological failure.

Designation of the community of patients was performed by self-identification. Scheduled caste community is marginalised in the traditional Hindu caste hierarchy and, therefore, suffers social and economical exclusion and disadvantage [21]. Scheduled tribe community is generally geographically isolated with limited economical and social contact with the rest of the population [21]. Scheduled castes and scheduled tribes are considered socially disadvantaged communities and are supported by positive discrimination schemes operated by the Government of India [22].

Clinical staging was performed following WHO guidelines for HIV disease in children [20].

In HIV infected children <5 years, the CD4 lymphocyte percentage has generally been preferred for monitoring the immune status because of the variability of the CD4 cell count during the first years of life [18]. However, an analysis of the HIV Paediatric Prognostic Markers Collaborative Study (HPPMCS) demonstrated that the CD4 percentage provides little or no additional prognostic value compared with the CD4 cell count in children [23]. Therefore, the immune status of children was calculated using the 12-month risk of AIDS used by the HPPMCS, which uses the CD4 cell count and the age of children to calculate the level of immunodeficiency (i.e., high 12-month risk of AIDS indicates low CD4 cell counts for the age and *vice versa*) [24]. Because of the small number of older children included in the HPPMCS, children older than 12 years were considered to be 12 years old to calculate the 12-month AIDS risk [25].

### Statistical analysis

To compare the effectiveness of NNRTI-based ART (NNRTI group) *versus* LPVr-based ART followed by switch to NVP-based ART after achieving virological suppression (LPVr induction-NVP maintenance group), we performed two analyses. For the first analysis, we studied the proportion of children who achieved virological suppression (HIV-RNA <400 copies/ml) during the first year of ART. Secondly, we performed a time-to-event analysis from ART initiation to virological failure among those children who achieved virological suppression and had available viral loads after one year of ART.

Continuous variables were compared using the rank sum test and categorical variables were compared using the χ^2^ test. Univariate and multivariable analysis of factors associated with initial virological suppression were performed using logistic regression. Univariate and multivariable analysis of factors associated with time to virological failure after initial virological suppression were performed using Cox proportional hazard regression. To include all children in the multivariable models, missing values were imputed using multiple imputations by chained equation assuming missing at random [26].

In a sensitivity analysis to minimize the effect of confounding and obtain an unbiased estimate of the treatment effect, differences in baseline characteristics of the two groups were balanced using propensity score methods to estimate the average treatment effect. To include non-linear effects and interactions, propensity scores were estimated via boosted models using the “twang” package in the R statistical computing environment (R Foundation for Statistical Computing, Vienna, Austria) [27]. To select the optimal interation of generalized boosted models, we set to minimize the means of the absolute standardized difference [28]. The propensity scores were used to estimate the inverse probability of treatment weights (IPTW) [28]. As two variables had missing values, multiple imputations were performed to obtain twenty complete datasets. IPTW were computed for each dataset and then, we calculated the average of the IPTWs [29]. These sampling weights were used to compare the initial virological suppression and the time to virological failure of the two groups using robust variance to account for the weighted nature of the sample [30].

Except for the estimation of propensity scores, the statistical analysis was performed using Stata Statistical Software (Stata Corporation. Release 12.1, College Station, Texas, USA).

## Results

During the study period, 466 children started ART. 55 children were excluded because they died or were lost to follow up before having a viral load determination (10 in the LPVr induction-NVP maintenance group and 45 in the NNRTI group). Although previous exposure to NVP was not an exclusion criterion, none of the caretakers of children included in the study referred previous exposure to NVP.

Eighty-six children who had viral load determinations after one year of ART but not during the first year were not included in the first analysis. Therefore, 325 were included in the analysis of virological suppression during the first year of ART, 83 in the LPVr induction-NVP maintenance group and 242 in the NNRTI group (205 were on NVP and 37 were on efavirenz). In the LPVr induction-NVP maintenance group, the median duration of LPVr before switch to NVP was 213 days (interquartile range 180–250). Differences between the two groups are presented in **Table 1**. The proportion of children in WHO clinical stage 3 or 4 was higher in the LPVr induction-NVP maintenance group. Children in the NNRTI group used more commonly liquid formulation and stavudine in their ART regimens.

**Table 1:** Characteristics of 325 HIV infected children initiating antiretroviral therapy in Anantapur, India.

	NNRTI group n = 242	LPVr group n = 83	P-value
Categorical variables	N (%)	N (%)	?^2^
Gender			0.346
Male	134 (55.4)	41 (49.4)	
Female	108 (44.6)	42 (50.6)	
Disadvantaged community			0.738
No	188 (77.7)	63 (75.9)	
Yes	54 (22.3)	20 (24.1)	
Living with parents			0.064
No	72 (29.8)	16 (19.3)	
Yes	170 (70.2)	67 (80.7)	
WHO clinical stage			0.012
1–2	152 (62.8)	39 (47)	
3–4	90 (37.2)	44 (53)	
Baseline viral load			0.973
<100,000 copies/ml	20 (45.5)	9 (45)	
>100,000 copies/ml	24 (54.5)	11 (55)	
Missing values (N)	198	63	
NRTIs			<0.001
d4T+3TC	148 (61.2)	29 (34.9)	
AZT+3TC	86 (35.5)	52 (62.7)	
ABC+3TC	8 (3.3)	2 (2.4)	
Liquid formulations			<0.001
No	181 (74.8)	79 (95.2)	
Yes	61 (25.2)	4 (4.8)	

Continuous variables	Median (IQR)	Median (IQR)	Rank sum
Age	7.8 (5.2–10.9)	8.9 (6.1–10.7)	0.173
12-month AIDS risk	3.7 (3.2–5.5)	3.6 (3.2–4.9)	0.481
Missing values (N)	4	0	

3TC, lamivudine; ABC, abacavir; AZT, zidovudine; IQR, interquartile range; LPVr, lopinavir boosted with ritonavir; NNRTI, non-nucleoside reverse transcriptase inhibitor; NRTI, nucleoside reverse transcriptase inhibitor.

74/83 (89.2%) of children in the LPVr induction-NVP maintenance group achieved virological suppression during the first year of ART *versus* 185/242 (76.4%) in the NNRTI group (p = 0.013; unadjusted odds ratio [OR] 2.53, 95% confidence interval [CI] 1.19–5.38). Univariate and multivariable analysis of factors associated with initial virological suppression are presented in **Table 2**. In the univariate analysis, the use of liquid formulations in the ART regimen was associated with lower probability of virological suppression. In the multivariable analysis with multiple imputation of missing values of the baseline viral load and the 12-month AIDS risk, female gender was associated with virological suppression (adjusted odds ratio [aOR] 2.06, 95% CI 1.08–3.91). The use of LPVr *versus* NNRTI was associated with higher probability of virological suppression (aOR 3.19, 95% CI 1.11–9.13). In sensitivity analyses, removing high baseline viral load from the model and imputing only missing values of 12-month AIDS risk (aOR 2.42, 95% CI 1.08–5.42) or using only complete cases (aOR 2.49, 95% CI 1.11–5.58) showed similar results. In the IPTW model, the use of LPVr was also associated with higher probability of virological suppression (OR 2.41, 95% CI 1.1–5.4), and the estimated risk difference was 12.2% (95% CI, 2.9–21.5).

**Table 2:** Factors associated with virological suppression (HIV-RNA <400 copies/ml) during the first year of antiretroviral therapy.

	OR (95% CI)	aOR (95% CI)†
Female gender	1.41 (0.81–2.45)	2.06* (1.08–3.91)
Age (years)	0.98 (0.91–1.06)	0.91 (0.81–1.02)
Disadvantaged community	0.67 (0.36–1.23)	0.44 (0.14–1.40)
Living with parents	1.34 (0.74–2.41)	1.16 (0.47–2.83)
WHO clinical stage 3–4	1.40 (0.80–2.46)	1.54 (0.75–3.17)
Baseline VL>100,0000 c/ml	0.39 (0.1–1.59)	0.24 (0.02–2.49)
NRTIs		
d4T+3TC	1 (reference)	1 (reference)
AZT+3TC	0.70 (0.41–1.21)	0.53 (0.27–1.03)
ABC+3TC	1.99 (0.24–16.24)	1.66 (0.17–16.08)
Use of liquid formulations	0.44* (0.24–0.81)	0.47 (0.21–1.05)
12-month AIDS risk (%)	1.00 (0.96–1.03)	1.01 (0.96–1.06)
LPVr vs. NNRTI	2.53* (1.19–5.38)	3.19* (1.11–9.13)

3TC, lamivudine; aOR, adjusted odds ratio; ABC, abacavir; AZT, zidovudine; CI, confidence interval; LPVr, lopinavir boosted with ritonavir; NNRTI, non-nucleoside reverse transcriptase inhibitor; NRTI, nucleoside reverse transcriptase inhibitor; VL, viral load.

*P-value<0.05;

† To include all patients in the multivariable model, missing values of baseline viral load and 12-month AIDS risk were imputed using chained equations (64 children had complete data available).

**Figure 1:**
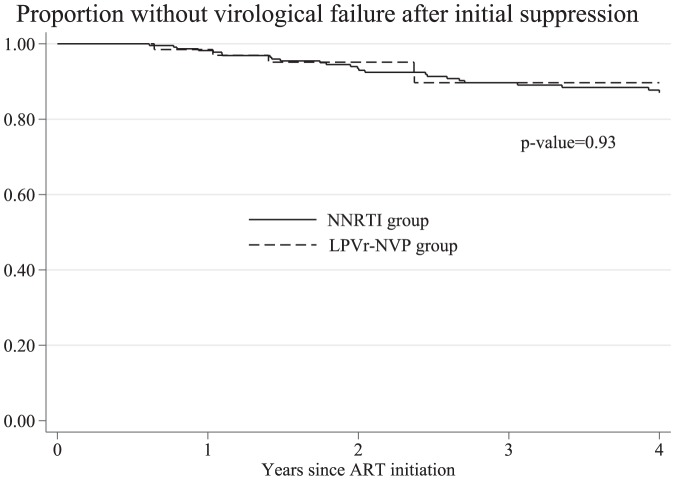
Proportion of children without virological failure after initial virological suppression over time. LPVr-NVP, ritonavir boosted lopinavir-based regimen followed by switch to nevirapine-based regimen; NNRTI, non-nucleoside reverse transcriptase inhibitor-based regimen.

In the second analysis of time to virological failure, we included 292 children who achieved virological suppression and had viral load determination after one year of ART, 66 in the LPVr induction-NVP maintenance group and 226 in the NNRTI group (197 were on NVP and 29 were on efavirenz). Differences between the two groups were similar to the ones found in the analysis of initial virological suppression (**Table 3**). The Kaplan-Meier estimates of the incidence of virological failure showed no significant differences between the two groups (**Figure 1**). Univariate and multivariable analysis of factors associated with initial virological failure are presented in **Table 4**. In the multivariable analysis with multiple imputation of missing values of the baseline viral load and the 12-month AIDS risk, we did not find statistically significant differences in time to virological failure between the LPVr induction-NVP maintenance group and the NNRTI group (adjusted hazard ratio [aHR] 1.18, 95% CI 0.36–3.81). In sensitivity analyses, removing high baseline viral load from the model and imputing only missing values of 12-month AIDS risk (aHR 1.00, 95% CI 0.35–2.91) or using only complete cases (aHR 0.98, 95% CI 0.34–2.83) showed similar results. In the IPTW model, we also did not find statistically significant differences in time to virological failure between the LPVr induction-NVP maintenance group and the NNRTI group (HR 1.48, 95% CI 0.54–4.01; p-value = 0.443).

**Table 3:** Characteristics of 292 HIV infected children who achieved virological suppression (HIV-RNA <400 copies/ml) after antiretroviral therapy initiation in Anantapur, India.

	NNRTI group	NVP switch group	P-value
	n = 226	n = 66	
Categorical variables	N (%)	N (%)	?^2^
**Gender**			0.316
**Male**	122 (54)	31 (47)	
**Female**	104 (46)	35 (53)	
**Disadvantaged community**			0.717
**No**	176 (77.9)	50 (75.8)	
**Yes**	50 (22.1)	16 (24.2)	
**Living with parents**			0.036
**No**	75 (33.2)	13 (19.7)	
**Yes**	151 (66.8)	53 (80.3)	
**WHO clinical stage**			0.003
**1–2**	151 (66.8)	31 (47)	
**3–4**	75 (33.2)	35 (53)	
**Baseline viral load**			0.456
**<100,000 copies/ml**	22 (57.9)	8 (47.1)	
**>100,000 copies/ml**	16 (42.1)	9 (52.9)	
**Missing values (N)**	188	49	
**NRTIs**			<0.001
**d4T+3TC**	154 (68.1)	22 (33.3)	
**AZT+3TC**	71 (31.4)	43 (65.2)	
**ABC+3TC**	1 (0.4)	1 (1.5)	
**Liquid formulations**			0.017
**No**	185 (81.9)	62 (93.9)	
**Yes**	41 (18.1)	4 (6.1)	

Continuous variables	Median (IQR)	Median (IQR)	Rank sum
**Age**	7.9 (5.5–10.4)	8.3 (6.1–10.4)	0.595
**12-month AIDS risk**	3.8 (3.2–5.5)	3.6 (3.3–4.9)	0.453
**Missing values (N)**	8	0	

3TC, lamivudine; ABC, abacavir; AZT, zidovudine; IQR, interquartile range; NNRTI, non-nucleoside reverse transcriptase inhibitor; NRTI, nucleoside reverse transcriptase inhibitor, NVP, nevirapine.

**Table 4:** Factors associated with virological failure (HIV-RNA >1000 copies/ml) in children who achieved initial virological suppression.

	HR (95% CI)	aHR (95% CI)†
Female gender	1.00 (0.52–1.93)	1.19 (0.52–2.73)
Age (years)	1.10 (0.99–1.22)	1.07 (0.94–1.23)
Disadvantaged community	0.30* (0.09–0.97)	0.32 (0.09–1.12)
Living with parents	0.88 (0.44–1.76)	1.03 (0.44–2.42)
WHO clinical stage 3–4	1.36 (0.70–2.63)	1.77 (0.69–4.57)
Baseline VL>100,0000 c/ml	0.24 (0.01–5.74)	0.12 (0.00–7.88)
AZT+3TC vs. others	0.85 (0.42–1.73)	0.70 (0.30–1.68)
Use of liquid formulations	0.52 (0.18–1.48)	0.61 (0.19–1.90)
12-month AIDS risk (%)	1.02 (0.97–1.07)	1.06 (0.97–1.16)
NVP switch vs. NNRTI	0.96 (0.36–2.53)	1.18 (0.36–3.81)

3TC, lamivudine; aHR, adjusted hazard ratio; AZT, zidovudine; CI, confidence interval; NNRTI, non-nucleoside reverse transcriptase inhibitor; NRTI, nucleoside reverse transcriptase inhibitor; NVP, nevirapine; VL, viral load.

*P-value<0.05;

† To include all patients in the multivariable model, missing values of baseline viral load and 12-month AIDS risk were imputed using chained equations (55 children had complete data available).

Among 71 children in the NNRTI group and 12 in the LPVr induction-NVP maintenance group who had virological failure, genotypic resistance testing was available in 44 children of the NNRTI group and in three of the LPVr induction-NVP maintenance group (**Table 5**). The median time to virological failure was 395 days (interquartile range [IQR] 279–731) in the NNRTI group and 245.5 days (IQR, 211.5–556) in the LPVr induction-NVP maintenance group. While in the LPVr induction-NVP maintenance group only one child had two-class resistance (NNRTI and NRTI), 36 (82%) children in the NNRTI group had two-class resistance.

**Table 5:** Drug resistance in children with virological failure.

ARV Resistances	NNRTI group (count)	LPVr-NVP group (count)
No resistance	0	1
NNRTI	8	0
3TC	0	1
NNRTI+3TC	18	1
NNRTI+3TC+other NRTIs	18	0

3TC, lamivudine (mutation M184V); ARV, antiretroviral; LPVr-NVP, ritonavir boosted lopinavir-based regimen followed by switch to nevirapine-based regimen; NNRTI, non-nucleoside reverse transcriptase inhibitor; NRTI, nucleoside reverse transcriptase inhibitor;

## Discussion

In this cohort study using routine clinical data of children three years and older from a resource-limited setting, the use of LPVr-based ART was associated with an increased probability of initial virological suppression and a subsequent switch from LPVr to NVP was not associated with a higher risk of virological failure compared with children starting NNRTI-based ART. The results of this study are in contrast with the 2013 guidelines of the WHO, which recommend NNRTI-based regimens for first line ART in children three years and older [20]. If our findings are confirmed in other settings, the results of this study could have important public health implications to help reduce virological failures among children starting ART in developing countries.

The higher proportion of children achieving initial virological suppression with LPVr induction therapy is in accordance with an international multisite clinical trial in children younger than three years [31]. In this clinical trial, children in the NVP group had higher risk of virological failure, and those who experienced virological failure had more drug resistances [31]. In our study, we observed fewer resistances in the LPVr induction-NVP maintenance group too, but the number of children with available drug resistance testing was small. While protease inhibitors have a relatively high barrier to the development of resistance, there are several single-gene mutations that lead to NNRTI resistance. NNRTI resistance occurs early after starting ART, when viral load and viral replication are high and, therefore, the chances of development of NNRTI mutations are also higher [32]. Once the viral load is low, the risk of developing NNRTI mutations is considerably reduced.

After virological suppression, the incidence of virological failure was similar in both groups, indicating that children switched from LPVr to NVP-based regimens did not have higher risk of virological failure than those children who started ART with NNRTI-based regimens and achieved virological suppression. However, in our study none of the children was exposed to NVP for prevention of vertical transmission, so switching from LPVr to NVP in previously exposed children may require more intense virological monitoring [33].

Viral load monitoring is not readily available in developing countries, so the implementation of this induction-maintenance strategy might be problematic where viral load is not available. However, the 2013 WHO guidelines, which recommend using viral load monitoring in HIV patients on ART, and the commercialization of new low-cost and simple viral load technologies might lead National HIV programmes in low- and middle-countries to scale-up viral load monitoring in the near future [20], [34]. On the other hand, the majority of children in our study received six to nine months of LPVr-based ART before switch to NVP-based regimens, resulting in lower rates of virological failure and resistance. In setting where viral load is not available, induction therapy with LPVr-based ART during six or nine months followed by switch to NVP-based ART instead of ART initiation with NNRTI-based regimens could be beneficial, but new studies are needed to confirm this hypothesis.

The study has some limitations. Our results reflect the “real life” of HIV infected children in a resource-limited setting. However, unlike clinical trials, observational studies can be biased due to unknown confounders. The selection of treatment was not randomized, so factors not included in the multivariable analyses might have influenced the outcomes of study. In addition, we did not have complete data for all cases; particularly many children had missing values of baseline HIV viral load. Nevertheless, we performed extensive sensitivity analyses, which showed similar results with different statistical methods. Our findings need to be confirmed by observational studies performed in other settings or, ideally, by a randomized clinical trial.

## Conclusions

In a large cohort of HIV infected children three years and older from a resource-limited setting and without previous exposure to NVP, starting ART with LPVr-based regimens was associated with higher probability of virological suppression than starting with NNRTI-based regimens. Once virological suppression was achieved, children switched from LPVr to NVP-based treatment did not have a higher risk of virological failure than children who started NNRTI-based ART and achieved virological suppression. If these results are confirmed in other settings, this LPVr induction- NVP maintenance strategy could help reduce virological failures among HIV infected children three years and older starting ART.
